# Smells Like Anthelmintic Resistance—Gastrointestinal Prevalence, Burden and Diversity in Dogs from Portugal

**DOI:** 10.3390/pathogens13090799

**Published:** 2024-09-14

**Authors:** Cláudia Luís Batista, Ricardo Cabeças, Cláudio Araújo-Paredes, Maria Aires Pereira, Teresa Letra Mateus

**Affiliations:** 1 Escola Superior Agrária, Instituto Politécnico de Viana do Castelo, 4990-706 Ponte de Lima, Portugal; enfvetclaudia@gmail.com; 2 Centro de Investigação Vasco da Gama (CIVG), Escola Universitária Vasco da Gama (EUVG), 3020-210 Coimbra, Portugal; ricardo.cabecas@euvg.pt; 3 CISAS—Center for Research and Development in Agrifood Systems and Sustainability, Escola Superior Agrária, Instituto Politécnico de Viana do Castelo, Rua Escola Industrial e Comercial de Nun’Àlvares, 4900-347 Viana do Castelo, Portugal; cparedes@esa.ipvc.pt; 4 proMetheus, Research Unit in Materials, Energy and Environment for Sustainability, Instituto Politécnico de Viana do Castelo, 4900-347 Viana do Castelo, Portugal; 5 Instituto Politécnico de Viseu, Escola Superior Agrária de Viseu, Campus Politécnico, 3504-510 Viseu, Portugal; 6 Global Health and Tropical Medicine, GHTM, Associate Laboratory in Translation and Innovation towards Global Health, LA-REAL, Instituto de Higiene e Medicina Tropical, IHMT, Universidade NOVA de Lisboa, UNL, Rua da Junqueira 100, 1349-008 Lisboa, Portugal; 7 CERNAS-IPV Research Centre, Instituto Politécnico de Viseu, Campus Politécnico, Repeses, 3504-510 Viseu, Portugal; 8 EpiUnit—Instituto de Saúde Pública da Universidade do Porto, Laboratory for Integrative and Translational Research in Population Health (ITR), Rua das Taipas, nº 135, 4050-091 Porto, Portugal; 9 Veterinary and Animal Research Centre (CECAV), UTAD, Associate Laboratory for Animal and Veterinary Sciences (AL4AnimalS) Quinta de Prados, 5000-801 Vila Real, Portugal

**Keywords:** Ancylostomatidae, Mini-FLOTAC, one health, Taeniidae, *Toxocara* spp., *Trichuris* spp., zoonosis

## Abstract

Anthelmintic resistance has been documented worldwide, but few cases have been reported in dogs. Shelter dogs are a great opportunity to study intestinal helminths and assess their potential resistance to anthelmintic compounds. With these two goals in mind, 361 faecal samples were collected from dogs in 18 municipalities in Portugal, from 19 shelters and 11 private tutors. These samples were analysed using the Mini-FLOTAC before and 13 days after deworming. The percentage of faecal egg count reduction was calculated. Parasitic forms were identified in 22.4% of the samples collected: *Trichuris vulpis* (10.5%), *Toxocara canis* (8.0%), members of the family Ancylostomatidae (6.4%), *Toxascaris leonina* (0.8%), *Dipylidium caninum* (0.6%) and members of the family Taeniidae (0.3%). The first three nematode parasites showed the highest intensity of infection (2900, 1325, and 1265 eggs per gram, respectively). In the second faecal collection, parasites were present in 20.8% of the samples. The anthelmintics febendazole + pyrantel embonate + praziquantel and praziquantel + fenbendazole were ineffective for *T. vulpis* and members of the family Ancylostomatidae in 16.2% and 6.9% of the samples, respectively. The parasites identified are all potentially zoonotic. This study is the first to suggest a potential resistance of *T. vulpis* to anthelmintics.

## 1. Introduction

Intestinal parasites, both helminths and protozoa, are commonly found in domestic animals, and their transmission from dogs to humans constitutes a potential zoonotic risk across the globe [1]. The strict cohabitation between dogs and humans sharing the same living areas increases this zoonotic risk [2]. Among intestinal helminths, both *Ancylostoma* spp. and *Toxocara* spp. are the most important nematodes affecting dogs [3] and are also a concern for public health [4,5,6].

Rescue shelters represent a good observatory for the evaluation of the pathogens that circulate among dog populations [2]. Dogs admitted to shelters may carry intestinal parasites that could pose serious risks to other animals, shelter staff, and visitors [7]. Nevertheless, companion animals placed in shelters are typically under the supervision of veterinarians, receive proper vaccinations, and are given dewormers to avoid the spread of parasites [1].

Although close contact with dogs is not considered to be the most relevant risk for the transmission of intestinal helminths, the presence of their infective elements in the environment should always be considered a zoonotic risk factor [2,8]. Parasite transmission and distribution are most often influenced by climatic, geographical, cultural, and socio-economic factors. Furthermore, the level of sanitary conditions, absence of veterinary supervision, and lack of awareness concerning zoonotic diseases contribute to the spreading of parasite transmission [3,9].

The inappropriate massive use of antiparasitic compounds to treat and prevent intestinal helminthoses often leads to the development of resistance [10]. Multidrug-resistant hookworms have been identified recently by Castro and Kaplan, especially in dog kennels [11]. Resistant parasites that survive the initial treatments transmit their resistance genotype(s) to the next generation, and within a few, the initially small resistant subpopulation begins to expand, ultimately leading to treatment failure [12]. Selection pressure for resistance is influenced by the genetics and biology of the parasite, the size of the parasite subpopulation that is not selected by the antiparasitic compound (“refugia”), the host–parasite relationship, and management practices, including the selection of antiparasitic compound, dosage, and planning of antiparasitic treatment [10].

However, anthelmintic treatments are the most important component of the systematic and effective prevention of clinical helminthoses [12]. The major classes of anthelmintics used for pets are the benzimidazoles, the nicotinic agonist, and the macrocyclic lactones [13]. Some classes of anthelmintics exhibit different efficacies against various stages or several helminth species [14].

Pyrantel was the first anthelmintic to be used massively in tablets to treat intestinal nematodes in dogs and might be, therefore, expected to be the first drug to fail as a result of anthelmintic resistance [12]. Pyrantel has a spectrum against only hookworms and ascarids [9,15]. Benzimidazoles have a spectrum against whipworms but have poor efficacy against immature stages. For hookworms, both pyrantel and benzimidazoles have reasonably good activity against developing intestinal larval stages, although benzimidazoles have better activity against immature ascarids [7,9]. For pets, a product combining febantel and pyrantel that exhibits a synergistic efficacy has been used for decades, unintentionally establishing a propitious situation that conserves susceptibility to these compounds [12].

Due to low levels of resistance, benzimidazoles can temporarily sterilise female nematodes without removing them. Mebendazole and fenbendazole are also effective against some of the common cestodes. Praziquantel demonstrates a marked anthelmintic activity against a wide range of adult and larval cestodes and trematodes [9].

Although no new ways have been found to control resistance to anthelmintics, it is necessary to create effective strategies to minimise its impact. This requires an understanding of the biology of parasites, how they become resistant, and what alternative strategies of control exist [10].

Nonetheless, the low sensitivity of faecal examination techniques hinders parasitological diagnosis and the assessment of anthelmintic efficacy. Indeed, any tests that rely on visual detection of the parasite in faeces are subject to certain implicit limitations. For instance, microscopic examination of faecal specimens is unable to detect prepatent infections or those involving a single-sex nematode. Furthermore, ideal flotation techniques may vary across different stages of parasites. However, due to time constraints and the implementation of standardised protocols, a single method is frequently used for all faecal tests [8].

Therefore, standard diagnostic and treatment protocols for pet endoparasites can be significantly limited by treatment failure, the low sensitivity of faecal examination techniques, and especially the possibility of reinfection from the same or new environmental sources. While there is evidence that animals are capable of developing an immune response to parasitic infections, protection from future challenges is uncertain. It can also be more difficult to diagnose infections with subsequent exposure due to reduced faecal egg counts and attenuated clinical signs [16].

Little is known about the recurrence rates of common endoparasites in companion animals [12,16]. Drug resistance against nematodes is considered uncommon in companion animals. Potential reasons for the rare finding of anthelmintic resistance development in dog intestinal helminths, compared to livestock, include major epidemiological and biological differences, an overall lower anthelmintic treatment frequency, significantly different husbandry settings, and a smaller parasite population size that leads to a comparatively low genetic diversity in the parasite populations [12]. However, investigations are beginning to appear regarding drug resistance in dog intestinal nematodes; still, publications on this topic are limited. Recently, in the USA, several cases of *Dipylidium caninum* (Linnaeus, 1758) resistant to praziquantel have been reported [17], as well as of hookworms to multiple drugs [11].

There are concerns about the impact that frequent anthelmintic applications may have in terms of increasing resistance selection pressure. Therefore, this study has a two-fold aim: to assess the prevalence of intestinal helminth parasites in dogs from dog shelters (public and private) and dogs with owners from the north and centre of Portugal and to assess the suspected resistance of anthelmintics based on the faecal egg count reduction test (FECRT).

## 2. Materials and Methods

### 2.1. Samples and Data Collection

**Figure 1:**
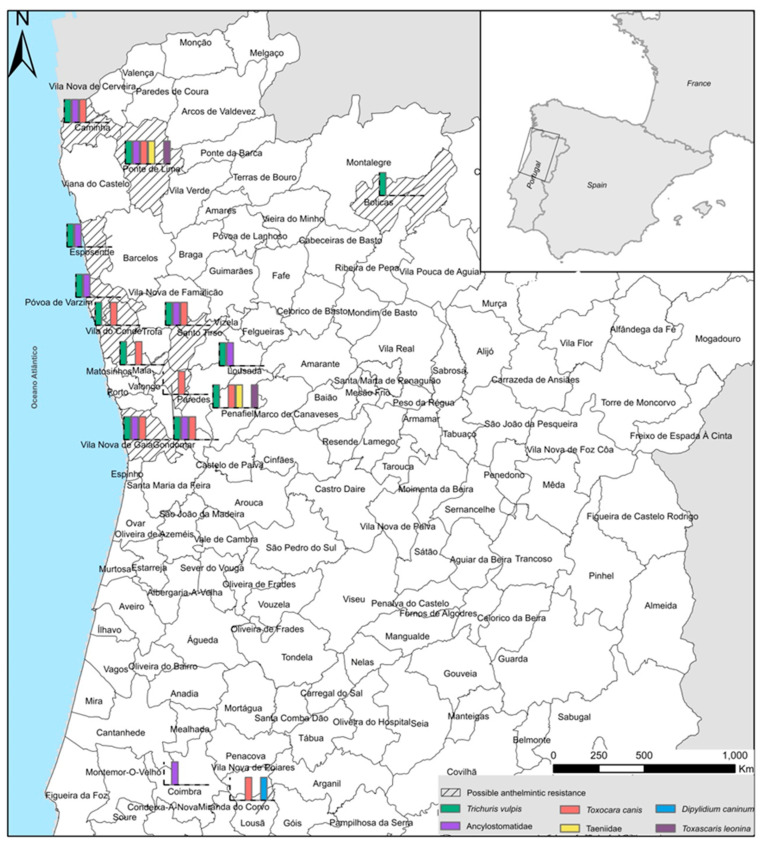
Geographical distribution and diversity of intestinal helminths identified and possible anthelmintic resistance in dogs’ helminths in different municipalities of the north and centre of Portugal.

Fourteen public shelters, five private shelters, and eleven private owners accepted our invitation to participate in this study. Between May 2020 and February 2021, faecal samples were collected from asymptomatic dogs of different breeds (pure or mixed breed), genders, and ages. According to age, dogs were classified as less than six months old, six months to one year, one to four years old, four to ten years old, and more than ten years old. Other information was gathered, such as lifestyle (indoor/outdoor), consumed diets (commercial/others), stool consistency (normal/abnormal), reproductive status (fertile/non-fertile), when was the last deworming (less than three months, three to eight months, nine to twelve months, more than twelve months), which drugs were used (febantel + praziquantel + pyrantel; fenbendazole + pyrantel embonate + praziquantel; praziquantel + fenbendazole; milbemycin oxime + praziquantel; pyrantel + oxantel + praziquantel; epsiprantel + pyrantel), if they lived alone (without contact with other animals in the same compartment) or with other animals, and if the canids were vaccinated (rabies or rabies and other vaccines) or not. These animals were kept in individual boxes, in public/private shelters, or had owners. The samples were collected in the north and centre of Portugal (municipalities of Anadia, Boticas, Caminha, Coimbra, Esposende, Gondomar, Lousã, Lousada, Maia, Paredes, Penafiel, Ponte de Lima, Porto, Póvoa de Varzim, Santo Tirso, Valongo, Vila do Conde, and Vila Nova de Gaia) (Figure 1), given that these are regions with few epidemiological studies and because this study was performed during the COVID-19 confinements and, in Portugal, there were travel restrictions. The first collection was made on the day before the dogs were dewormed, and whenever parasitic forms were found, a second collection was made thirteen days after deworming. The samples were collected immediately after defecation, stored in plastic bags, identified, transported at 4 °C, and processed within 48 h through coprological methods in the laboratory of the Escola Superior Agrária of the Instituto Politécnico de Viana do Castelo (ESA—IPVC), Portugal.

The suspected resistant helminth infection was assessed considering the FECRT. The formula used to calculate the FECRT percentage was adapted from that suggested by Castro and Kaplan (2020) [11] as follows: faecal egg count reduction (FECR) percentage = (number of parasite eggs on the day before treatment—number of parasite eggs in treated animals)/number of parasite eggs on the day before treatment) × 100. These authors suggest performing two faecal egg counts before and two after deworming, but in the present study, only one before and one after was performed as there were national travel restrictions due to the COVID-19 confinements. If the FECR is greater than 95%, the treatment is considered effective—between 90–95% is suggestive of reduced efficacy and suspected resistance, between 75–89% is suggestive of resistance, and less than a 75% reduction is indicative of resistance [11].

### 2.2. Copromicroscopic Analyses

Each faecal sample was macroscopically checked for tapeworm proglottids and adult roundworms and then analysed using the Mini-FLOTAC technique for egg detection and quantification using a Fill-FLOTAC Kit and a conventional optical microscope. Two flotation solutions were used—saturated sodium chloride (NaCl) and zinc sulphate (ZnSO_4_)—according to the instructions reported in the original description by Cringoli et al. [18]. Eggs and oocysts were identified according to the descriptions of Zajac and Conboy [6].

### 2.3. Statistical Analysis

The statistical analysis was conducted using R Statistical Software^®^ version 3.6.2 [19]. Data were expressed as percentages and mean ± standard deviations. Bivariate associations were conducted using Pearson’s chi-square test for the categorical data. The values of *p* < 0.05 were considered statistically significant.

## 3. Results

### 3.1. Sample Characterisation

Faecal samples were collected from dogs kept in 19 different shelters and from 11 private owners. The characterisation of the 361 samples collected and the presence/absence of parasites are presented in Table 1.

Most of the samples came from public shelters (81.4%). The dogs were mostly of mixed breed (93.1%), male (58.5%), and castrated (54.6%), with 75.9% being between 4 and 10 years old and 58.7% were vaccinated. A percentage of dogs lived outdoors (51.5%), and 62.3% lived with no contact with other animals. Most of the dogs (80.1%) ate commercial diets, and 65.7% of the faeces were abnormal in consistency. The most used anthelmintic was fenbendazole + pyrantel pamoate + praziquantel (35.2%), and 39.6% of the dogs had been dewormed 3 to 8 months before the first faecal collection.

Regarding the origin of the samples, the prevalence was higher in the samples from public shelters (23.5%), but considering the aptitude, the higher prevalence came from pets. Considering the breed, gender, and reproductive status, the presence of parasites was more frequent in mixed breeds (23.5%), those that were male (23.7%), and those that were non-fertile (26.4%), respectively. Concerning other variables, such as lifestyle, coexistence, diet, and faecal consistency, the prevalence of parasites was higher in the samples from dogs kept indoors (26.9%), alone (26.7%), that ate commercial diets (22.8%), and which faeces had an abnormal consistency (23.6%). Dogs with ages between six months and one year and more than 10 years old had a higher parasite prevalence (33.3%). The presence of parasites was identified in 42.1% of the animals that were not dewormed for at least one year. Concerning the dewormers used, the presence of parasites was identified in 28.6% of the animals that were dewormed with pyrantel + oxantel + praziquantel, as well as in dogs that have not been dewormed. Regarding vaccine status, animals that were not vaccinated had a higher total parasite prevalence (31.1%).

### 3.2. Prevalence and Burden of Parasites

The faecal samples of 361 dogs were collected in 18 municipalities of the north and centre of Portugal (Figure 1), and in 81 of them, helminths were found; therefore, the overall prevalence was 22.4% (95% CI: 18.24–27.10%) (Table 2).

The highest prevalence was found in public shelter dogs (23.1%). Seven species/genera/families of intestinal parasites were detected, including nematodes, cestodes, and protozoa. *Toxocara canis* (Werner, 1782) had a higher prevalence in dogs with owners (15.0%). *Trichuris vulpis* (Froelich, 1789) and members of the Ancylostomatidae family were mostly found in private shelters (7.4% and 3.7%, respectively) and public shelters (11.2% and 8.8%, respectively) (Table 3).

Overall, the most frequently observed parasite was *T. vulpis* (10.5%), followed by *T. canis* (8.0%) and members of the Ancylostomatidae family (6.4%). *Toxocara canis* had a mean elimination of 257 eggs per gram (EPG), followed by *Toxascaris leonina* (Linstow, 1902) (220 EPG), *T. vulpis* (191 EPG), and members of the Ancylostomatidae family (146 EPG) (Table 4).

The parasites and parasite associations found in the three different groups of dog faecal samples are presented in Table 5. Regarding the macroscopic analysis, adult parasites were found in five (5/361, 0.01%) samples, and in one of them, no eggs were found in the microscopic analysis.

Concerning single infections, *T. canis* had a higher prevalence in dogs with owners (15.0%). *Trichuris vulpis* was more frequently found in private shelters, while the members of the Ancylostomatidae family were mostly found in public shelters. Cestodes were only found in public shelters. Most mixed infections were found in public shelters, but one association of parasites (*T. vulpis* + Ancylostomatidae) had a higher prevalence (5.0%) in dogs with an owner.

### 3.3. Variables Association

Parasite prevalence was higher in dogs that were alone (26.7%) than in dogs that lived with other animals (15.4%) (*p* = 0.02). No significant associations (*p* > 0.05) were found between the prevalence of the most common parasites and the diet or the faecal consistency.

The occurrence of *T. canis* was significantly associated with the season of the year (*p* = 0.025), with the highest prevalence occurring between the months of January and March (12.1%) and September and December (11.8%). The prevalence of *T. canis* was higher in dogs with owners (15.0%; *p* = 0.04), while the prevalence in public shelter dogs was 6.1%. The occurrence of *T. canis* was significantly associated with age (*p* = 0.04), with the highest prevalence occurring in dogs less than one year of age (33.3%), following dogs that were more than ten years old (16.7%).

The time of the ‘last deworming’ was statistically associated with the presence of *T. vulpis* (*p* = 0.005) and members of the Ancylostomatidae (*p* = 0.037) and Taeniidae families (*p* = 0.021). The highest prevalence for these parasites occurred in dogs that were dewormed more than one year ago (26.3%, 21.1%, and 5.3%, respectively). The occurrence of *T. vulpis* was statistically associated (*p* = 0.022) with the lifestyle of the dogs, with a higher parasite prevalence in indoor dogs (14.3%) than in outdoor dogs (7.0%). Vaccination against rabies was statistically significantly associated with the prevalence of *T. vulpis* (*p* = 0.001) and members of the Ancylostomatidae family (*p* < 0.001), having a prevalence higher in unvaccinated dogs (18.2% and 14.2%, respectively) than in vaccinated dogs (4.4% for both).

### 3.4. Anthelmintic Resistance

A total of 4 out of 81 dogs, where samples with parasites were identified, were adopted, so thirteen days later, a second faecal collection was performed in 77 dogs for FECRT. In 16 (20.8%) of the faecal samples, parasitic forms were found again.

Anthelmintics were apparently effective against the following different parasite taxa: *T. canis*, *T. leonina*, members of the Taeniidae family, *D. caninum*. *Trichuris vulpis*, and members of the Ancylostomatidae family were found to be resistant (Table 6).

Anthelmintics febendazole + pyrantel embonate + praziquantel and praziquantel + fenbendazole were found to have less efficacy against *T. vulpis* and Ancylostomatidae (Table 7).

## 4. Discussion

In this study, the overall prevalence of intestinal parasites was 22.4% and seven different families/genera/species of parasites were found in sheltered dogs and dogs with owners from 18 municipalities from the north and centre of Portugal. The prevalence of intestinal parasites in some Portuguese studies was variable, ranging from 20.6% to 72.5%. A prevalence higher than those of the present study was observed; for example, in household and shelter dogs from the Alentejo region (39.2%) [1], shelter dogs from the north region (57.2%) [20], farm dogs from the centre region (58.8%) [21], environmental dogs’, farm dogs’ and hunting dogs’ samples from the north region (63.17%) [22], and stray dogs from the Área Metropolitana de Lisboa (72.5%), Portugal [23]. A slightly low total parasite prevalence was observed in healthy and gastrointestinal-diseased dogs from the north region (20.6%) of Portugal [24]. Several factors related to the study’s design may influence the diversity of parasitic forms found as well as their prevalence, namely the origin of the sampled dogs, the regions studied, and the parasitological methods employed.

Regarding the origin of the dogs, the parasite prevalence was similar in samples from public shelters (23.1%) and dogs with an owner (22.5%) and was lower in private shelters (11.1%). Studies comparing different dog populations (stray dogs, owned dogs, kenneled dogs, and shelter dogs) found that shelter dogs are especially prone to parasitic infection [7]. The high density of animals in a restricted area, especially in poor management conditions, contributes to environmental contamination, thereby increasing the risk of infection [2]. Furthermore, it is known that shelter animals usually face many stressors, such as overcrowding or isolation, unusual environments, limited physical activity, and a poor diet, among others, favouring the establishment and dissemination of intestinal parasitic infections, especially those transmitted by direct contact or ingestion of contaminated food, water, soil, and from licking contaminated surfaces [7].

In this study, the prevalence of infection in dogs with owners was higher compared with other studies carried out in Portugal [1,24] as well as in other countries [25]. In Spain, dogs with owners showed a high risk of helminth infection due to inadequate deworming [9]. In a survey in France, low deworming levels were related to suboptimal owner education [26]. In a similar survey carried out in Portugal, most dog owners were unaware of how often they dewormed their dogs and how to do it. Non-compliance of pet owners who were less prepared to treat their pets against parasites or were not aware of the correct way of treatment [27] may justify the prevalence of intestinal parasites in the dogs with owners registered in this study.

Differences in the overall parasitic prevalence among shelters have already been reported [2]. The low parasite prevalence registered in private shelters compared with public shelters may be related to shelter management, particularly with the accomplishment of effective control programs, including proper husbandry practices, hygiene measures (environmental cleaning and disinfection), use of appropriate parasitological diagnostic methods in housed animals—particularly in new arrivals—and appropriate use of deworming compounds [2,7]. In some of the shelters studied in this work, dogs were dewormed only once upon admission, while in other shelters, they were dewormed regularly, three or four times per year, which may justify the differences in parasite prevalence among shelters.

The results of this survey showed that single infections were more frequent than mixed infections and that helminths were more prevalent than protozoa, which is in agreement with the results recorded in the previous studies performed in Portugal [20,21,22,28] and in other countries [2,16].

Concerning single infections, *T. vulpis* (10.5%) was the most frequent parasite, followed by *T. canis* (8.0%) and members of the Ancylostomatidae family (6.4%). In some Portuguese surveys, the members of the Ancylostomatidae family were more prevalent than *Trichuris* spp. [1,20,21,22], while in other countries, such as in the USA [16] and Italy [2], the most prevalent was *Trichuris* spp. The higher prevalence of *T. vulpis* and *T. canis* is probably due to the high resistance of trichurid and ascarid eggs in the environment for a long time. Moreover, it seems that trichurid reinfection increases with age [16]. Furthermore, according to the *European Scientific Counsel Companion Animal Parasites* (2020) [4], to be effective, dogs infected with *T. vulpis* must be dewormed more than once in brief intervals, which was not the case with any sheltered dog or owned dog in this study, which may contribute to the high prevalence observed. Also, these three parasites showed a high burden of infection in some dogs, with a maximum egg shedding per sample of 2900, 1325, and 1265 EPG, respectively, which represents a potential health risk for shelter workers, owners, and dogs.

*Toxascaris leonina* was the less frequently found ascarid in this study, as other studies confirm [1,22,24,29]. The prevalence of cestodes was even lower since *D. caninum* was identified only in one sample, and members of the Taeniidae family were identified in two samples. In general, the detection of Taeniidae eggs in faecal samples by routine microscopy suffers from low sensitivity [2,30]. Eggs are detected when free in the faeces by flotation techniques. Generally, however, the eggs are passed from the host contained in the tapeworm segments. Therefore, faecal flotation tends to be a poor indicator of infection status [6]. Also, the probability of finding members of the Taeniidae family in kennels is very low, given the nature of the diets. In fact, most of the dogs in this study were fed commercial dog food, justifying the low prevalence of Taeniidae; as well, *Cystoisospora* spp. was identified only in two samples. Visual microscopy poses even more significant limitations for the detection of protozoan parasites, as they are typically represented by very small-sized cysts or oocysts in the faeces, which are difficult to reliably detect visually [7]. The low prevalence of the members of the Taeniidae family, *D. caninum,* and *Cystoisospora* spp. detected in this study is in accordance with the values previously found in surveys where few or none were found in Portugal [21,22,28] as well as in other European countries [2,9,31].

In this study, dogs that had contact with other animals, such as indoor or outdoor cats, other dogs, farm animals, or the excrement of wild animals, revealed a significantly lower prevalence of intestinal parasites than dogs that were alone. This finding is not consistent with other studies since it is expected that a higher prevalence of parasites in dogs that coexist with other animals would be found [30]. Antolová et al. (2004) [32] have pointed out the crucial role of red foxes, dogs, and small mammals in the circulation and maintenance of parasite eggs in suburban and rural regions. However, in this study, most parasitised dogs that were alone belonged to a public shelter, living on a cement floor with contact with free-living rats. Also, the last deworming for most of these kennelled dogs was unknown, while dogs that coexist with other animals were frequently dewormed. These facts may explain the higher prevalence of intestinal parasites in dogs that were alone.

The prevalence of *T. canis* was significantly higher in dogs with an owner (15.0%) than in public shelters (4.1%) and private shelters (0.0%). Several factors may contribute to the increased prevalence of *T. canis* infection in dogs with owners. *Toxocara* eggs are very resistant to adverse environmental conditions and may remain infective for years [7,14]. Most pet owners enjoy a high amount of off-leash walking time [33], and others maintain their dogs chained on a soil area, which may explain the increased frequency of infection. A survey in Lithuania indicates that the elimination/excretion of *T. canis* eggs one month after anthelmintic treatments was significantly higher in the chained dogs mostly kept on soil compared with dogs maintained in pens, suggesting the intestinal passage of unembryonated eggs, previously ingested by coprophagy [14]. Since there are no practical methods for reducing environmental egg burdens, prevention of soil contamination is the most important tool [13]. Hygienic measures include the appropriate disposal of faeces and the destruction of expelled worms [12,13]. The low prevalence of *T. canis* in shelters may be related to the facilities/husbandry characteristics (impermeable floors/paved areas) that allow correct cleaning and disinfection and the absence of scarce dog outdoor activity, which limits soil contact. Indeed, parasite transmission is more provable in unpaved areas, where soil provides protection for parasites and makes processes of cleaning areas and disinfection ineffective [7].

The occurrence of *T. canis* was significantly associated with low temperatures, occurring between the months of January and March and September and December. This result is in accordance with a study in the USA where roundworm prevalence was consistent each year (2012–2018), with the highest seasonal prevalence occurring from December to January [34].

The prevalence of *T. canis* was significantly higher in dogs less than one year of age (33.3%) and in dogs more than ten years old (16.7%), which is consistent with the literature [2,16,32,35,36]. The increased prevalence in young animals is probably related to the diversity of transmission routes, namely vertical (transplacental and transmammary) and horizontal transmission through the ingestion of embryonated eggs from the environment or the ingestion of larvae via vertebrate and/or invertebrate paratenic hosts [4,13]. The high prevalence of *T. canis* in old animals may be related to the loss of acquired immune protection [37].

*Trichuris vulpis* was more frequently identified in indoor dogs rather than outdoor dogs. Generally, dogs that live outdoors or have unrestricted access to the soil and environment are at greater risk of acquiring parasites [4,38]. The higher prevalence of *T. vulpis* in indoor dogs might be related to the fact that outdoor dogs are more exposed to parasites; therefore, owners might feel the need to deworm them more often—or maybe these indoor dogs lived in a contaminated environment that promoted constant reinfection. Regardless, responsible ownership of pets includes regular health controls with faecal diagnostics and deworming, accompanied by regular testing for efficacy [4].

Dogs that were vaccinated (against rabies and rabies and others) had a lower prevalence of *T. vulpis* (4.4% and 6.8%, respectively) and members of the Ancylostomatidae family (4.4% and 1.4%, respectively) than unvaccinated dogs. In a Spanish survey about endoparasite infection risk and deworming frequencies, 84.83% of dog owners relied on their veterinarian’s recommendations [9]. In a similar Portuguese study, the majority of pet owners that attend veterinary clinics gave endoparasiticides and ectoparasiticides to their pets [39]. Commonly, when pet owners attend veterinary clinics/hospitals to deworm their pets, veterinarians also advise them to vaccinate their pets or vice-versa [27].

To assess anthelmintic resistance, faecal samples of the sampled dogs were analysed thirteen days after deworming, and the anthelmintic resistance was evaluated based on the percentage of FECRT calculation [5,9,11]. The time between treatment and the second egg count should vary according to the anthelmintic group. Concerning benzimidazoles, it should be 7 to 10 days, for nicotinic agonists from 3 to 7 days, and for macrocyclic lactones, 14 days [11]. In a Lithuanian investigation, egg counts were conducted before treatment and at 9, 10, 16, and 21 days after treatment [14].

*Toxocara canis*, *T. leonina*, members of the Taeniidae family, and *D. caninum* were completely eliminated by the anthelmintics used. However, *T. vulpis* eggs remained present in eight faecal samples, and members of the Ancylostomatidae family’s eggs were identified in three samples after deworming. This recurrence was found in five different public shelters and one pet with an owner. Several investigations have already demonstrated drug resistance, including multiple drug resistance to *A. caninum* [5,11,17,40,41,42].

However, evaluating the anthelmintic efficacy in Ancylostomatidae based on the FECRT is unreliable since hookworms can modulate their individual egg production according to the intestinal adult parasite population [40]. For this reason, it is imperative that in vitro assays, such as the egg hatch and larval development assays that allow the identification of drug-resistant phenotypes, and the genetic tests used to characterise drug-resistant genotypes [41,43], must be included in future investigations of anthelmintic resistance involving this nematode. Moreover, immature larval stages of *A. caninum* possess the ability to migrate and encyst in somatic tissues, either remaining there or ultimately finding their way into the gastrointestinal tract [17,40]. During pregnancy, encysted larvae in somatic tissues will reactivate and migrate to the mammary gland. These encysted or migrating larvae have the potential to repopulate the gastrointestinal tract. Pyrantel—like other anthelmintics that are not very well absorbed from the gastrointestinal tract—are mainly competent in the adult stages and will not affect somatic larvae, which may lead to *A. caninum* treatment being challenging [17].

In this study, the drug combinations that revealed some degree of inefficacy against *A. caninum* were febendazole + pirantel embonate + praziquantel, praziquantel + fenbendazole and febantel + praziquantel + pyrantel. According to the literature, these compounds have a spectrum against hookworms. In the registration studies, febantel demonstrated an efficacy > 99% [44,45,46], fenbendazole > 98% [47], and pyrantel demonstrated a slightly variable efficacy, with a mean across the studies of approximately 94%, where more than half of those studies yielded >99% [48]. Furthermore, Vienažindienė et al. (2021) [14] described that the combination of febantel + pyrantel + praziquantel is highly effective (94–98%) against *A. caninum*. Nevertheless, several studies have already identified *A. caninum* as being resistant to albendazole, moxidectin, or a combination of febantel–pyrantel–moxidectin [41], and pyrantel pamoate, fenbendazole, and milbemycin oxime [43].

In this study, 10.5% of the dogs identified with a latent infection by *T. vulpis* returned to eliminate parasite eggs after deworming. The percentage of FECR of *T. vulpis* was indicative or suggestive of resistance with the combinations febendazole + pyrantel embonate + praziquantel, febantel + praziquantel + pyrantel, and praziquantel + fenbendazole. To the best of our knowledge, there are no reports of drug resistance to *T. vulpis* in dogs. According to the Companion Animal Parasite Council (CAPC), febantel + pyrantel pamoate + praziquantel in a single administration and fenbendazole for three consecutive days is approved for the treatment of *T. vulpis* infections in dogs. However, to achieve control, treatment can be administered once a month for three months. According to the CAPC guidelines, dogs infected with *T. vulpis* received inappropriate treatment since they were dewormed only once. Furthermore, anthelmintic drugs were administered by the shelter workers as recommended by the veterinarian or were administered by the owner, and due to lack of material or poor logistics, the dogs may not have been weighed before anthelmintic treatment, and thus, a low dosage could have been administered.

Despite our results raising the suspicion of anthelmintic resistance, the detected therapeutic inefficacy may be due to other causes, such as inadequate nutrition, rapid reinfection, inappropriate anthelmintic choice, administration of an incorrect dose below the recommended ones, faulty administration, presence of developing or arrested larvae unaffected by the anthelmintic treatment, reduced anthelmintic efficiency in some disease conditions [49], or even parasite genetics and biology, as well as host–parasite relationships [10]. Moreover, this study performed only one FEC before and after deworming and not two as suggested [11] to improve the accuracy of the FECRT. That is also why this study may only suggest the presence of anthelmintic resistance.

It has been observed in livestock that frequent usage of the same group of anthelmintic may result in the development of anthelmintic resistance and there is evidence that resistance develops more rapidly in regions where animals are dewormed regularly [50]. This might be problematic for places such as shelters. According to Shalaby [50], treating animals simultaneously with two drugs from different anthelmintic classes is one method of preventing the development of anthelmintic resistance.

## 5. Conclusions

There were some limitations of this study that related to the confinement imposed by the COVID-19 pandemic, which compromised the adhesion of dog and shelter owners to this study, and some methodology limitations, namely the lack of information available on the weights of the animals. The impossibility of collecting faeces over three consecutive days to avoid false-negative results (related to the intermittent excretion of eggs), as well as the impossibility of adjusting the interval between the two faecal collections according to the anthelmintic group used, were also limitations; however, despite these, this study is the first to evaluate potential anthelmintic resistance in intestinal helminths in dogs from Portugal. Further investigations need to be conducted in the future to confirm and better understand the extent of anthelmintic resistance. It is worth highlighting that the overall parasite prevalence was high (22.4%), being that all the parasites identified as zoonotic or potentially zoonotic. This highlights the risk to public health and underlines the need for a one-health approach.

## Figures and Tables

**Table 1:** Characterisation of the samples collected and presence/absence of parasites (in number and percentage).

Variable	Total (*n*)	Total (%)	Presence (*n*)	Presence (%)
**Origin**				
Public shelters	294	81.4	69	23.5
Dogs with owner	40	11.1	9	22.5
Private shelters	27	7.5	3	11.1
**Aptitude**				
Shelter	322	89.2	73	22.7
Pet	22	6.1	6	27.3
Guard	17	4.7	2	11.8
**Months**				
May	17	4.7	3	17.6
June	66	18.3	13	19.7
July	81	22.4	23	28.4
August	44	12.2	9	20.5
September	24	6.6	4	16.7
October	59	16.3	14	23.7
November	2	0.6	0	0.0
December	26	7.2	4	15.4
January	1	0.3	1	100
February	41	11.4	9	22.0
**Breed**				
Mixed breed	336	93.1	79	23.5
Pure breed	25	6.9	2	8.0
**Gender**				
Male	211	58.5	50	23.7
Female	134	37.1	28	20.9
NI	16	4.4	4	25.0
**Reproductive status**				
Non-fertile	197	54.6	38	19.3
Fertile	163	45.1	43	26.4
NI	1	0.3	0.0	0.0
**Age class**				
<6 months	2	0.6	0	0.0
6 months–1 year	6	1.7	2	33.3
1–4 years	61	16.9	12	19.7
4–10 years	274	75.9	61	22.3
>10 years	18	5.0	6	33.3
**Last deworming (in months)**				
<3	32	8.9	7	21.9
3–8	143	39.6	32	22.4
9–12	51	14.1	5	9.8
>12	19	5.3	8	42.1
NI	116	32.1	29	25.0
**Lifestyle**				
Outdoor	186	51.5	33	17.7
Indoor	175	48.5	47	26.9
**Coexistence**				
Alone	225	62.3	60	26.7
With other animals	136	37.7	21	15.4
**Diet**				
Commercial	289	80.1	66	22.8
Others	72	19.9	14	19.4
**Faecal consistency**				
Abnormal	237	65.7	56	23.6
Normal	124	34.3	24	19.4
**Vaccine status**				
Vaccinated	212	58.7	35	16.5
Not vaccinated	148	41.0	46	31.1
NI	1	0.3	0	0.0
**Dewormers compounds used**				
Fen + Pyr pamoate + Pra	127	35.2	34	26.8
Pra + Fen	85	23.5	17	20.0
Feb + Pra + Pyr	79	21.9	13	16.5
None	35	9.7	10	28.6
Eps + Pyr	19	5.3	3	15.8
Mil + Pra	9	2.5	2	22.2
Pyr + oxantel + Pra	7	1.9	2	28.6

NI—not identified; Fen—fenbendazol; Pyr—pyrantel; Pra—praziquantel; Feb—febantel; Eps—epsiprantel; Mil—milbemycin oxime.

**Table 2:** Presence and absence (in number and percentage) of intestinal parasites in public shelter dogs, private shelter dogs, and owner dogs.

Presence of Parasites	Public Shelter		Dogs with Owner		Private Shelter		Total	
	*n*	%	*n*	%	*n*	%	*n*	%
Presence	69	23.1	9	22.5	3	11.1	81	22.4
Absence	225	76.9	31	77.5	24	88.9	280	77.6
Total	294	100	40	100	27	100	361	100

**Table 3:** Prevalence (%) of parasites found in 361 dog faecal samples from the three different groups.

Parasites	Dogs with Owner (*n* = 40) (%)	Private Shelters (*n* = 27) (%)	Public Shelters (*n* = 294) (%)
*Trichuris vulpis* (Froelich, 1789)	7.5	7.4	11.2
*Toxocara canis* (Werner, 1782)	15.0	0.0	5.8
Ancylostomatidae	5.0	3.7	8.8
*Toxascaris leonina* (Linstow, 1902)	0.0	0.0	1.0
Taeniidae	0.0	0.0	0.7
*Cystoisospora* spp.	0.0	0.0	0.7
*Dipylidium caninum* (Linnaeus, 1758)	0.0	0.0	0.3

**Table 4:** Prevalence (%), mean, and maximum (Max) of eggs or oocysts per gram (E/OPG), and parasitic burden of dogs’ intestinal parasites from north and centre of Portugal.

			No. Samples per Parasitic Burden Interval			
Parasites	Prevalence (%)	Mean E/OPG ± SD	5–100 E/OPG	100–500 E/OPG	>500 E/OPG	Max
*Trichuris vulpis*	10.5	191 ± 528.4	27	8	3	2900
*Toxocara canis*	8.0	257 ± 332.7	10	11	3	1325
Ancylostomatidae	6.4	146 ± 260.5	19	8	2	1265
*Toxascaris leonina*	0.8	220 ± 152.4	1	2	0	340
*Cystoisospora* spp.	0.6	25 ± 0	2	0	0	25
*Dipylidium caninum*	0.6	15 ± 0	1	0	0	15
Taeniidae	0.3	5 ± 0	2	0	0	5

**Table 5:** Parasites and association of parasites (in percentage) found in three different groups of dog faecal samples.

Parasites	Dogs with Owner (*n* = 40) (%)	Private Shelters (*n* = 27) (%)	Public Shelters (*n* = 294) (%)
*Toxocara canis*	15.0	0.0	4.1
*Trichuris vulpis*	2.5	7.4	6.5
Ancylostomatidae	0.0	3.7	5.8
*Toxascaris leonina*	0.0	0.0	0.7
Taeniidae	0.0	0.0	0.7
*Cystoisospora* spp.	0.0	0.0	0.3
*D. caninum*	0.0	0.0	0.3
*Trichuris vulpis* + Ancylostomatidae	5.0	0.0	3.1
*Trichuris vulpis* + *Toxocara canis*	0.0	0.0	1.7
*Toxascaris leonina* + *Cystoisospora* spp.	0.0	0.0	0.3
*Toxocara canis +* Ancylostomatidae + *Cystoisospora* spp.	0.0	0.0	0.3

**Table 6:** FECR percentage per identified parasite.

FECR (%)	*Trichuris vulpis* (*n* = 37)	Ancylostomatidae (*n* = 29)	*Toxocara canis* (*n* = 21)	*Toxascaris leonina* (*n* = 2)	Taeniidae (*n* = 2)	*Dipylidium caninum* (*n* = 1)
>95	75.7	86.2	100	100	100	100
90–95	2.7	6.9	-	-	-	-
75–89	5.4	-	-	-	-	-
<75	16.2	6.9	-	-	-	-

**Table 7:** FECR percentage of *T. vulpis* and Ancylostomatidae using different anthelmintics.

Efficacy	<75%		75–89%		90–95%		>95%	
Parasite/ Anthelmintics	*T. vulpis*	Ancylostomatidae	*T. vulpis*	Ancylostomatidae	*T.* *vulpis*	Ancylostomatidae	*T.* *vulpis*	Ancylostomatidae
**Feb +** **Pra + ** **Pyr (%)**	NA	NA	2.7 (*n* = 1)	NA	NA	NA	8.1 (*n* = 3)	17.2 (*n* = 5)
**Fen + Pyr embonate** **+ Pra (%)**	8.1 (*n* = 3)	3.4 (*n* = 1)	2.7 (*n* = 1)	NA	NA	3.4 (*n* = 1)	37.8 (*n* = 14)	44.8 (*n* = 13)
**Pra +** **Fen (%)**	5.4 (*n* = 2)	3.4 (*n* = 1)	NA	NA	2.7 (*n* = 1)	NA	29.7 (*n* = 11)	27.6 (*n* = 8)

NA—not applicable (*n* = 0).

## Data Availability

The original contributions presented in the study are included in the article, further inquiries can be directed to the corresponding author.
